# An Investigation of Biodiesel Production from Wastes of Seafood Restaurants

**DOI:** 10.1155/2014/609624

**Published:** 2014-10-07

**Authors:** Nour Sh. El-Gendy, A. Hamdy, Salem S. Abu Amr

**Affiliations:** ^1^Egyptian Petroleum Research Institute, P.O. Box 11727, Nasr City, Cairo, Egypt; ^2^Environmental Health Directorate, Ministry of Health, Gaza Strip, Palestine

## Abstract

This work illustrates a comparative study on the applicability of the basic heterogeneous calcium oxide catalyst prepared from waste mollusks and crabs shells (MS and CS, resp.) in the transesterification of waste cooking oil collected from seafood restaurants with methanol for production of biodiesel. Response surface methodology RSM based on D-optimal deign of experiments was employed to study the significance and interactive effect of methanol to oil M : O molar ratio, catalyst concentration, reaction time, and mixing rate on biodiesel yield. Second-order quadratic model equations were obtained describing the interrelationships between dependent and independent variables to maximize the response variable (biodiesel yield) and the validity of the predicted models were confirmed. The activity of the produced green catalysts was better than that of chemical CaO and immobilized enzyme Novozym 435. Fuel properties of the produced biodiesel were measured and compared with those of Egyptian petro-diesel and international biodiesel standards. The biodiesel produced using MS-CaO recorded higher quality than that produced using CS-CaO. The overall biodiesel characteristics were acceptable, encouraging application of CaO prepared from waste MS and CS for production of biodiesel as an efficient, environmentally friendly, sustainable, and low cost heterogeneous catalyst.

## 1. Introduction

Development of transesterification process for production of biodiesel as an alternative, green, and sustainable fuel has become an important issue due to diminishing fossil fuel reserves, rising crude oil price, and the stringent exhaust emission regulations [1].

Biodiesel produced by transesterification reaction can be catalyzed with alkali, acid, or enzyme, in which a primary alcohol reacts with the triglycerides of fatty acids form glycerol and esters. Triglyceride for biodiesel production comes from various sources edible and nonedible oil, waste and used oil, and fats [2]. The major hurdle of applicability of biodiesel is the operational cost of its production process, approximately 70–95% of the production cost arose from the feedstock. Homogenous chemical catalyst processes, including alkali or acid, are more practical compared with the enzymatic method. The use of acid catalysts has been found to be used for pretreating high free fatty acid feedstock but the reaction rates for converting triglycerides to methyl esters are very slow. Enzymes have shown good tolerance for the free fatty acid level of the feedstock but the enzymes are expensive. The use of homogenous basic catalytic process overcome the aforementioned problems but suffers some drawbacks: production of large amount of wastewater from washing process of catalyst residues and neutralization step, difficulty of the product separation and purification, and unreusability of the catalysts [3]. Heterogeneous catalytic process overcomes these drawbacks. Among the heterogeneous solid catalysts, calcium oxide CaO has attracted attention due to the elimination of neutralization step, high activity, being active in mild reaction conditions, long catalyst life time, low solubility in methanol, lack of toxicity, ability to withstand high temperatures, ease of recycling, low cost, and being abundantly available in nature as limestone and its performance for biodiesel production is comparable to several homogenous catalysts [4].

In Egypt, millions of liters of waste cooking oil WCO are discarded each year into sewage systems. Thus, it pollutes water streams, causing a lot of waste management problems and, consequently, adds to the cost of treating effluent. Solid wastes density in Egypt averages about 300 kg/m^3^, where 60% is organic wastes [5]. Thus, production of biodiesel from WCO using CaO prepared from organic wastes (e.g., eggshells, mollusks shells, crabs shells, etc.) would offer a triple-fact solution: economic, environmental, and waste management.

The aim of this work is to optimize biodiesel transesterification process using waste cooking oil and calcium oxide catalyst prepared from waste mollusks and crabs shells collected from seafood restaurants in an attempt to reach an effective process for practical, low cost industrial biodiesel production.

## 2. Materials and Methods

### 2.1. Materials

Pure calcium oxide as heterogeneous catalyst and methanol (AR Grade) were purchased from Fluka Chemical Corp., Gillingham, UK. Novozym 435 (*Candida antarctica* Lipase B) was a gift from Novozyme A/S, Bagsvaerd, Denmark, and was supplied as an immobilized enzyme on macroporous acrylic resin. Commercial Egyptian petro-diesel was obtained from a local fuelling station.

### 2.2. Collection and Preparation of Waste Frying Oil and Heterogeneous Catalyst

The waste frying oil WFO, mollusks, and crabs shells (MS and CS) were collected from local seafood restaurants and prepared according to El-Gendy et al. [3].

The WCO was characterized by high total acid number, density, and viscosity, recording 3 mg KOH/g oil, 0.9208 g/cm^3^, and 50 cSt, respectively, and its saponification and iodine value were 197 mg KOH/g oil and 119 mgI_2_/100 g oil, respectively. The WCO consists of ≈24.36, 36.35, 28.68, and 10.61% palmitic (C16:0), stearic (C18:0), oleic (C18:1), and linoleic (C18:2) acid, respectively.

### 2.3. Catalyst Characterization

The prepared catalysts were characterized according to El-Gendy et al. [3] using differential scanning calorimetric-thermal gravimetric analysis (DSC-TGA) and were performed by Q600 SDT Simultaneous DSC-TGA (New Castle, DE USA), a high-resolution X-ray diffractometer (XRD, PANalytical X'Pert PRO MPD, Netherland) coupled with Cu k*α* radiation source (*λ* = 1.5418 Å), Dispersive Raman spectrometer (BRUKER-SENTERRA, Germany) equipped with an integral microscope (Olympos), energy dispersive X-ray analysis (EDX, Oxford X-Max, England) conjugated with transmission electron microscope TEM (JEM 2100, Jeol, Japan), analytical Fourier transforms infrared (FT-IR, Perkin Elmer Spectrum One, USA) instrument, scanning electron microscope (SEM, JEOL-model JSM-53000, Japan). Particle sizer model Beckman Coulter Multisizer-3 (Nyon, Switzerland) was used for determination of particle size distribution. The specific surface area of the prepared biocatalysts was measured by Brunauer-Emmett-Teller BET method using low temperature N_2_ adsorption-desorption (NON A3200e, Quantachrome, USA). The samples were tested for pore volume and pore size distribution using Barrett-Joyner-Halenda BJH method. Temperature programmed desorption using CO_2_ as a probe molecules (CO_2_-TPD) was used to study basic properties of the prepared biocatalysts and it was done according to Viriya-empikul et al. [1].

### 2.4. Transesterification

The transesterification reactions were conducted in a laboratory-scale setup, according to El-Gendy et al. [3], and the biodiesel yield was calculated according to Boro et al. [6]. The activity of the prepared biocatalysts was compared with that of commercially available, most effective heterogeneous basic chemical catalyst CaO and immobilized enzyme Novozym 435.

### 2.5. Experimental Design and Statistical Analysis

Based on D-optimal design of experiments, twenty runs of experiments have been conducted for three levels of four independent variables: methanol : oil M : O (molar ratio; *A*), catalyst concentration (wt%; *B*), reaction time (min; *C*), and mixing rate (rpm; *D*), to study their effect on the % yield of the produced biodiesel at constant temperature 60°C. MATLAB 7.0 software (MathWorks, USA) was used for experimental design.

Once the experiments were preformed, the next step was to perform a response surface experiment to produce a prediction model to determine curvature, detect interactions among the design factors (independent variables), and optimize the process, that is, to determine the local optimum independent variables with maximum yield of biodiesel. The model used in this study to estimate the response surface is the quadratic polynomial represented by the following equation:

(1) $$\begin{array}{c} Y=\beta_{o}+\overset{n}{\underset{i=1}{\sum }}\beta_{i}x_{i}+\overset{n-1}{\underset{i=1}{\sum }}\overset{n}{\underset{j=i+1}{\sum }}\beta_{ij}x_{i}x_{j}+\overset{n}{\underset{i=1}{\sum }}\beta_{ii}x_{i}^{2}, \end{array}$$

where *Y* is the biodiesel yield (wt%), *n* is the number of factors, *β*_*o*_ is the intercept term, *β*_*i*_, *β*_*ij*_, and *β*_*ii*_ are the linear, interactive, and quadratic coefficients, respectively. *x*_*i*_'s are the levels of the independent variables (factors) under study.

The statistical software Design Expert 6.0.7 (Stat-Ease Inc., Minneapolis, USA) was used for regression and graphical analyses of the data obtained and statistical analysis of the model to evaluate the analysis of variance (ANOVA).

### 2.6. Physicochemical Characterization of the Produced Biodiesel

The produced biodiesel was tested for estimating and evaluating its fuel properties, using the standard methods of analysis for petroleum products, American Society for Testing and Materials ASTM standards methods [7]. The results were compared with the Egyptian standards for petro-diesel and European and American standards of biodiesel (EN14214 and D-6751, resp.) [8, 9].

All the properties were analyzed in two replicates and the final results given below were obtained as the average values.

## 3. Results and Discussion

### 3.1. Catalyst Characterization

**Figure 1:**
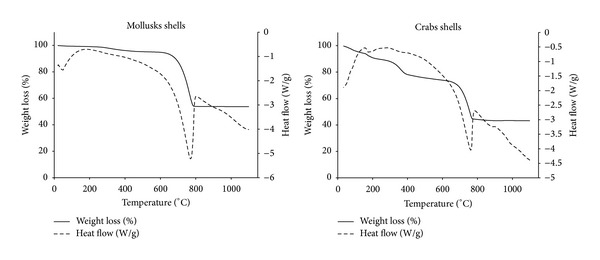
TGA/DSC curves.

The thermal transition during the calcination process of each collected waste shells was investigated with TGA/DSC.  Figure 1 shows the thermal analysis results along with the weight loss when the temperature was raised from room temperature to 1100°C.

The thermogravimetric analysis of MS shows that there was no significant decomposition occurred upon heating up to 600°C, recording weight loss of ≈5%. The dominant decomposition ≈41% weight loss occurred within 700–800°C (peaked at 778) and remained sustained thereafter up to 1100°C. The DSC curve supports the TGA curve, where the heat flow chart illustrates endothermic peak at 778°C. This reveals the production of new compound.

Upon calcination of CS, the TGA analysis reveals three phases of mass loss with a total weight loss of ≈56.77%. The first mass loss was observed at temperatures below 200°C, recording weight loss of ≈5% (peaked at 170°C). The second mass loss occurred within 250–400°C, recording weight loss of ≈20% (peaked at 320°C). The dominant decomposition with ≈30% weight loss occurred within 650–770°C (peaked at 750°C) and remained sustained thereafter up to 1100°C. The DSC curve shows an exothermic peak at 130°C. According to Boro et al. [6], this can be attributed to evaporation of water, crystallization, decomposition of organic components, or possible rearrangement in the structural arrangement within the compound itself. The DSC curve shows also a weak broad endothermic peak located at the range 320–370°C and another sharp endothermic peak at 753°C. The two endothermic peaks might indicate the decomposition of compounds and formation of a new one.

According to N. B. Singh and N. P. Singh [10], the weight loss might be attributed to the decomposition of CaCO_3_ through the loss of carbon dioxide CO_2_ and production of calcium oxide CaO and also due to the possible removal of absorbed water molecules which occurs according to the following dissociation equation:

(2) $$\begin{array}{c} \text{CaC}{\text{O}}_{3}\left(\text{s}\right)⟶\text{CaO}\left(\text{s}\right)+\text{C}{\text{O}}_{2}\left(\text{g}\right) \end{array}$$

It was observed that during calcination, the MS and CS turned completely pale grey and white, respectively. According to Engin et al. [11], this indicates that calcium carbonate CaCO_3_ is converted to CaO with elevating calcination temperatures.

**Figure 2:**
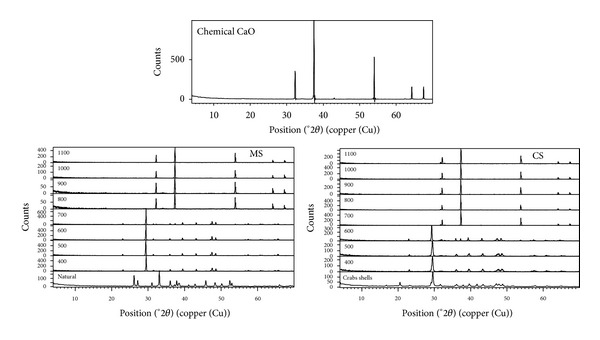
XRD patterns during calcination at different temperatures.

The XRD patterns of natural MS (Figure 2) are mainly aragonite CaCO_3_ (JCPDS card number: 024-0025) and upon calcination at 400°C, it was changed to calcite CaCO_3_ (JCPDS card number: 005-0586) and remained unchanged up to calcination at 600°C, while calcination at 700°C showed mixture of calcite CaCO_3_ (JCPDS card number: 005-0586) and lime CaO (JCPDS card number: 043-1001). The XRD patterns showed a pure crystalline CaO (JCPDS card number: 043-1001) for calcination temperatures 800–1100°C, while the XRD patterns of CS revealed that the natural CS is composed mainly of crystalline calcite CaCO_3_ (JCPDS card number: 005-0586) and also to a minor extent of monohydrocalcite CaCO_3_·H_2_O (JCPDS card number: 022-0147). Upon calcination at 400°C, CaCO_3_·H_2_O was lost and only calcite CaCO_3_ (JCPDS card number: 005-0586) detected and remained unchanged up to calcination at 600°C. The XRD patterns showed a pure crystalline calcia or burnt lime CaO (JCPDS card number: 004-0777) for calcination temperatures 700–1100°C. The obtained XRD patterns could explain the TGA/DSC flowcharts of CS, where the weak broad endothermic peak at the range of 320–370°C might be due to the loss of water and decomposition of CaCO_3_·H_2_O and the sharp one at 753°C might be due to the decomposition of CaCO_3_ and formation of CaO.

Narrow and high intense peaks of the calcinated MS and CS (at temperature ≥800 and 700°C, resp.) could define the well-crystallized nature of the prepared biocatalyst similar observation which was reported by Boey et al. [2] and Viriya-empikul et al. [12] on preparation of biocatalyst from waste mud crab shells (*Scylla serrata*) and waste mollusk shells for biodiesel production from palm olein oil.

The crystal size was calculated from the XRD data (Table 1), recording ≈42.15 nm for natural MS, which decreased upon calcination at 800°C, recording ≈38.77 nm. Similar observation was reported by Yoosuk et al. [13] who attributed this to the presence of water molecules during calcination of CaCO_3_ to CaO. But, the crystalline size of the uncalcinated CS was found to be ≈32.15 nm and it was slightly increased by calcination at 700°C, recording ≈32.82 nm. Borgwardt [14] and Liu et al. [15] reported that calcination of CaCO_3_ leads to formation and growth of CaO. When CaCO_3_ decomposes at high temperatures, small CaO grains formed and then the contact grains form necks and begin to grow resulting in an increase in the average grain size.

**Figure 3:**
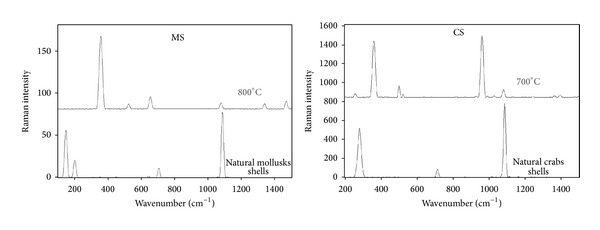
Raman spectra at different calcination temperatures.

Raman spectra (Figure 3) are consistent with the XRD patterns. In case of natural MS, the vibration bands at 155, 197, 650, 703, and 1086 cm^−1^ correspond to those of aragonite CaCO_3_. The vibration bands at 360, 1075, 1334, and 1463 cm^−1^ of calcined MS at 800°C correspond to pure lime CaO, while the vibration bands at 155, 282, 714, and 1087 cm^−1^ of natural CS correspond to calcite CaCO_3_. The high vibration bands at 360, 960, and 1075 cm^−1^ of calcined CS at 700°C correspond to pure burnt lime CaO.

EDX analysis confirmed also the results obtained from XRD and Raman. The EDX analysis (Table 2) revealed that the chemical composition of the shells was highly affected by calcination. The uncalcinated shells exhibit oxygen as the main component, 56.62 and 53.69%, wt%, while in calcinated shells at 800°C and 700°C, calcium represents the major component (62.32 and 49.89%, wt%) for MS and CS, respectively. Similar observation was reported by Viriya-empikul et al. [12] and Birla et al. [16], where the main component in the calcinated shells was calcium and other elements, for example, Na, Mg, and so forth, were found in trace amounts. Stoichiometrically, this is true, as in CaO, Ca is the main constituent (≈71%), while oxygen is the major one in CaCO_3_ (≈48%).

**Scheme 1:**
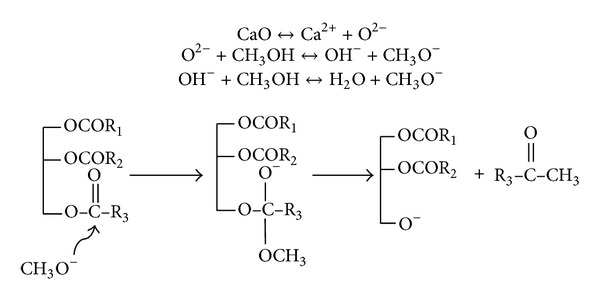


The basicity of the prepared CaO from MS and CS recorded 52.18 and 27 mmol CO_2_/g, respectively. This might affect the activity of the catalyst in the transesterification reaction, where the mechanism of CaO in transesterification reactions would be started by dissociation of CaO (Scheme 1). Then oxide anion attacks the methanol to form methoxide anion, which is why excess methanol is required to drive the reaction in the forward direction. The methoxide anion attacks the carbonyl carbon of the triglyceride to form a tetrahedral intermediate. The rearrangement of the intermediate molecule forms a mole of methyl ester and diglyceride. Another methoxide anion attacks the carbonyl carbon in the formed diglyceride, forming another mole of methyl ester and monoglyceride. Finally, a new methoxide anion attacks the monoglyceride, producing a total of three moles of methyl esters and a mole of glycerol.

**Figure 4:**
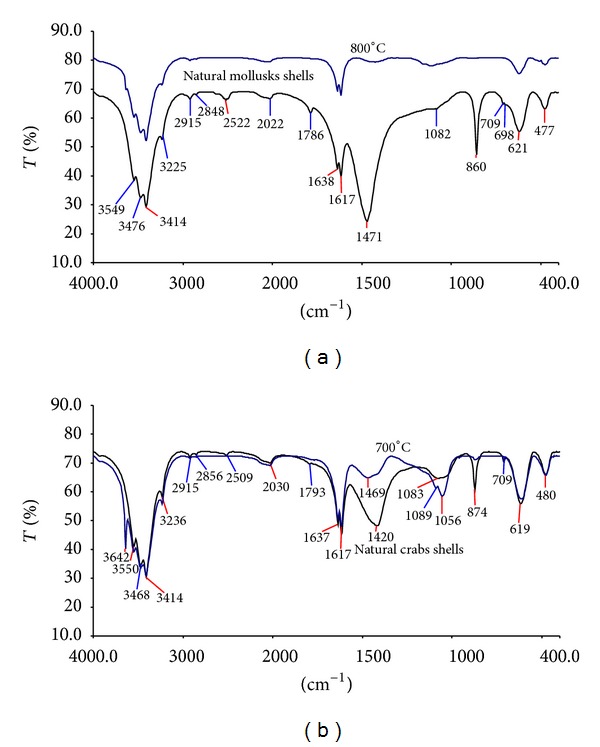
FTIR spectra of mollusks shells (a) and crabs shells (b) at different calcination temperatures.

The FTIR patterns of MS and CS with respect to calcination process (Figure 4) were nearly the same, showing major peaks around 1420–1471, 860–874, and 709 cm^−1^ in patterns of natural shells, which disappeared in that of calcinated shells. According to Engin et al. [11], these peaks are attributed to asymmetric stretch; out-of-plane bend and in-plane bend vibration modes for CO_3_^−2^ molecules. The strong sharp peak at 3643 cm^−1^ in uncalcinated CS confirms the XRD pattern and presence of CaCO_3_·H_2_O. Also, upon calcination, weak bands around 2509 and 1786–1793 cm^−1^ disappeared and new weak peak appeared at 1111 cm^−1^ for calcinated MS and 1089 and 1056 cm^−1^ for calcinated CS. The observed changes in IR patterns might indicate the complete transformation of CaCO_3_ to CaO. Similar observation was reported by Roschat et al. [17].

In the viewpoint of preparation time, energy consumption, and cost of catalyst preparation, the temperatures of 800°C and 700°C were selected as perfect calcination temperature to prepare biocatalysts from natural waste mollusks and crabs shells, respectively.

**Figure 5:**
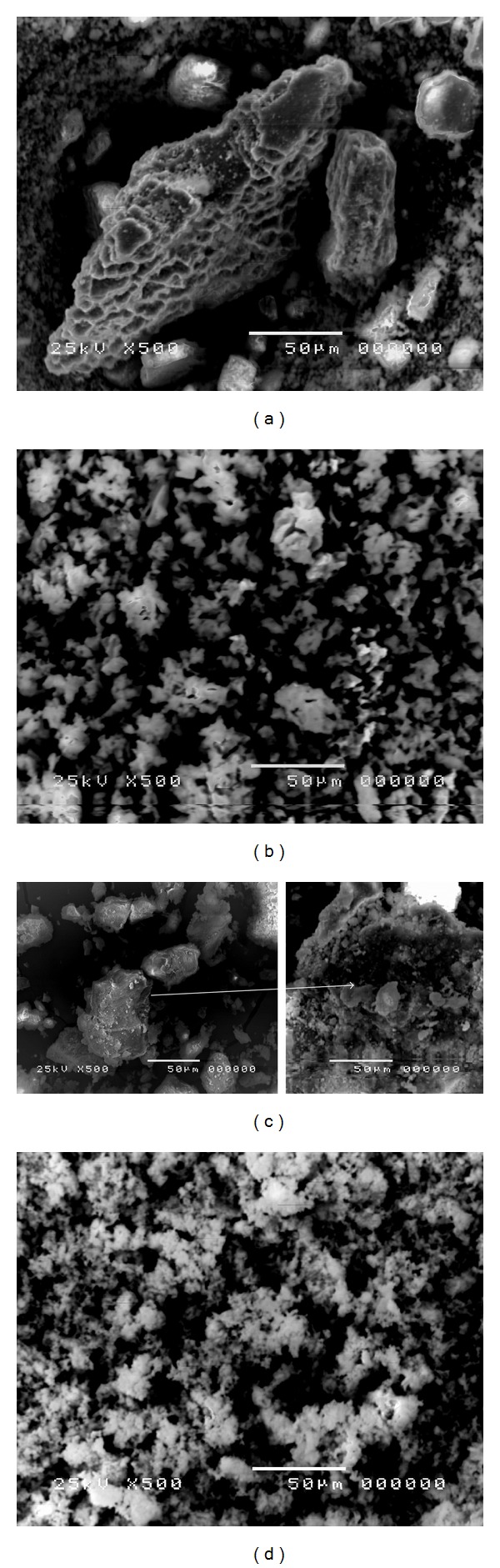
SEM images of natural mollusks shells (a), calcined mollusks shells at 800°C (b), natural crabs shells, (c) and calcined crabs shells at 700°C (d).

The morphology of natural and calcined shells was investigated by SEM at equal magnification of 500x. SEM micrograph of catalyst derived from MS (Figure 5(b)) shows that, upon calcination, the morphology of MS changed from layered bulky substances without any clear pores on its surface (Figure 5(a)) to porous particles of various sizes and shapes, with higher specific surface area. The SEM micrographs of the natural crabs shells (Figure 5(c)) showed bulky and nonuniform clustered substances with clear pores on its surface which transformed to relatively similar aggregates of porous smaller particles with higher specific surface area upon calcination at 700°C (Figure 5(d)). This porosity is probably due to the fact that a large number of gaseous water molecules are released upon the decomposition of CaCO_3_·H_2_O. Hu et al. [18] reported that the gaseous water molecules create high porosity in the catalyst, that is, to act as porogens.

The particles size distribution (Table 1) proved the SEM analysis (Figure 5), where a large part of the particles size distribution of the natural MS and CS was within the size range of 8.58–17.23 and 9.01–18.43 *μ*m, while the rest was within the range of 6.48–7.13 and 6.52–7.25 *μ*m, with overall average particles diameter of ≈11.07 and 10.27 *μ*m, respectively. But upon calcination at 800 and 700°C the particles size decreased, where a large part of the particles size distribution was in the range of 7.93–14.50 and 7.71–10.74 *μ*m and the rest was within the range of 6.37–6.80 and 6.38–6.81 *μ*m, with overall average particles diameter of ≈9.86 and 8.24 *μ*m, respectively. The smaller size of the grains and aggregates would provide higher specific surface areas. Because the prepared biocatalyst has relatively large particle sizes, it is easy to separate the catalyst from the products after the reaction, by filtration or centrifugation.

The BET surface area S_BET_ of the prepared catalysts (Table 1) recorded 12.63 and 43.73 m^2^/g for CaO prepared from MS and CS, respectively. This coincides with the SEM observation (Figures 5(b) and 5(d)). The BJH method was used for calculations of the pore size distributions (Table 1) and the prepared biocatalyst recorded total pore volume of ≈0.046 and 0.211 cm^3^/g, respectively, and the pore size, distributed between ≈0.83 and 17.18 nm and between 1.04 and 15.58 nm, with average pore diameter of 0.94 and 1.84 nm, respectively, indicating that most of the pore size distributions are found in the microporous range. According to Roschat et al. [17], a high porosity catalyst is a key requirement to achieve high conversion efficiency for heterogeneous process, thereby high surface area or high catalytic sites are necessary. Sharma et al. [19] also reported a high pore size to be desirable for better diffusion of reactants and product molecules. Thus, this would recommend the biocatalyst prepared from MS and CS for application in biodiesel production.

**Figure 6:**
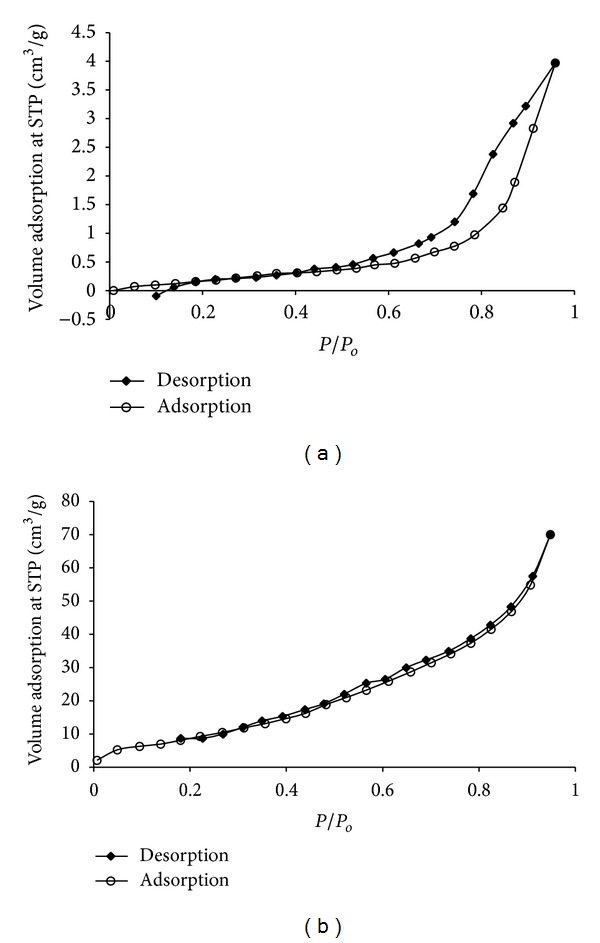
N_2_ adsorption-desorption isotherm of calcined MS at 800°C (a) and CS at 700°C.

The N_2_ adsorption-desorption isotherms of the prepared CaO from MS and CS are shown in Figures 6(a) and 6(b) and they are of type II isotherm (based on IUPAC's classification) with a low slope in the middle region of the isotherm and a desorption curve almost overlapping with adsorption curve. Applying BET equation, *P*/*P*_*o*_ = 0.9590 and 0.9482 (<1) and *C* = 7.152 and 17.012 (>1), respectively, indicating monolayer formation. The positive curvature at the lowest pressures indicates a distribution of adsorption energies. The isotherms have a hysteresis loop of H3 and slopping adsorption and desorption branches covering a large range of *P*/*P*_*o*_ with underlying type II isotherm.

### 3.2. Regression Model and Its Validation

The complete design matrix with experimental and predicted values of % yield of biodiesel using different CaO catalyst prepared from mollusks and crabs shells is presented in Table 3. Based on D-optimal design and experimental data, two second-order quadratic models for the applied catalysts have been predicted and can be given as follows:

(3) Y1=82.43−2.21A−0.98B+3.12C+0.80D+4.48A2+1.89B2−7.51C2−2.56D2−2.66AB−1.16AC+5.79AD+2.50BC+8.42BD+0.87CD,

(4) Y2=85.05+3.41A−3.28B−5.05C−0.6D−2.19A2+4.63B2+1.90C2+0.04D2+4.89AB−0.56AC+0.32AD−3.194BC−5.45BD+2.53CD,

where *Y*_1_ and *Y*_2_ are the biodiesel yields from transesterification processes using biocatalyst prepared from MS and CS, respectively.

Positive sign in front of the terms indicate synergetic effect, whereas negative sign indicates antagonistic effect.

**Figure 7:**
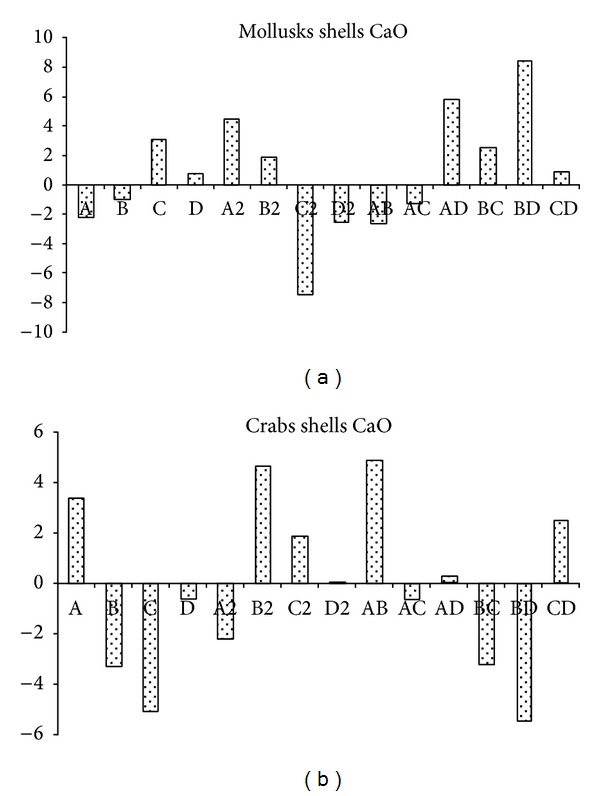
Pareto chart showing the effects of different independent variables on % biodiesel yield.

Pareto charts, which are very useful in design of experiments, were used in this work to make it much easier to visualize the main and interaction effects of all factors to the response variable that is biodiesel yield (Figure 7).

In case of using biocatalyst prepared from MS in the transesterification process, reaction time has a high positive impact on the reaction yield, followed by the mixing rate. But, M : O molar ratio has a high negative impact followed by catalyst loading. The interaction effects of M : O molar ratio and catalyst loading have a higher negative impact than M : O and reaction time on the biodiesel yield, while the interactive effect of catalyst loading and mixing rate expresses a very high positive impact on the biodiesel yield, coming after it the interaction between M : O ratio and mixing rate, catalyst loading, and reaction time, in a decreasing order, and the interactive effect of reaction time and mixing rate has a low positive impact on the reaction yield.

In case of using biocatalyst prepared from CS in the transesterification process, only the M : O molar ratio has positive impact; that is, increasing the M : O would increase the biodiesel yield. But, reaction time has a high negative impact followed by catalyst concentration and mixing rate, in a decreasing order; that is, their increase would lower the biodiesel yield. The positive interactive impact on the biodiesel yield was expressed by M : O and catalyst loading, reaction time, and mixing rate and M : O and mixing rate with a decreasing order. While the interaction between catalyst loading and mixing rate, catalyst loading, and reaction time and M : O and reaction time has negative impact on the biodiesel yield in a decreasing order.

The validity of the fitted models (3) and (4) was evaluated and their statistical significance was controlled by *F*-test. The analyses of variance (ANOVA) for the response surface full quadratic models are given in Tables 4 and 5. It can be indicated that the models are highly statistically significant at 95% confidence level, with *F* value of 940.12 and 99.58 with very low probability *P* value of <0.0001, respectively; that is, there is less than 0.01% chance that this error is caused by noise. The values of the determination coefficients, *R* ^2^ and *R*_adj_^2^, which measure the model fitting reliability for the models (3) and (4) were calculated to be (0.9996, 0.9986) and (0.9964 and 0.9864), respectively. This suggests that approximately 99.96% and 99.64% of the variance is attributed to the variables and indicated a high significance of the predicted models. Thus, only 0.04% and 0.36% of the total variations cannot be explained by the models, respectively, which ensures the good adjustment of the above models to the experimental data. Confirmation of the adequacy of the regression models was reflected also by the good agreement between experimental and predicted values of response variables as shown in Table 3, where the actual biodiesel yield for mollusks and crabs shells biocatalyst ranged from 71.80 to 92% and from 74 to 97% and there corresponding predicted values are 71.80 and 91.75% and 74 and 96.50%, respectively. “Adeq Precision” measures the signal-to-noise ratio. A ratio greater than 4 is desirable. The ratio of 102.01 and 36.452, respectively, indicated an adequate signal. These models are reliable and can be used to navigate the design space.

**Figure 8:**
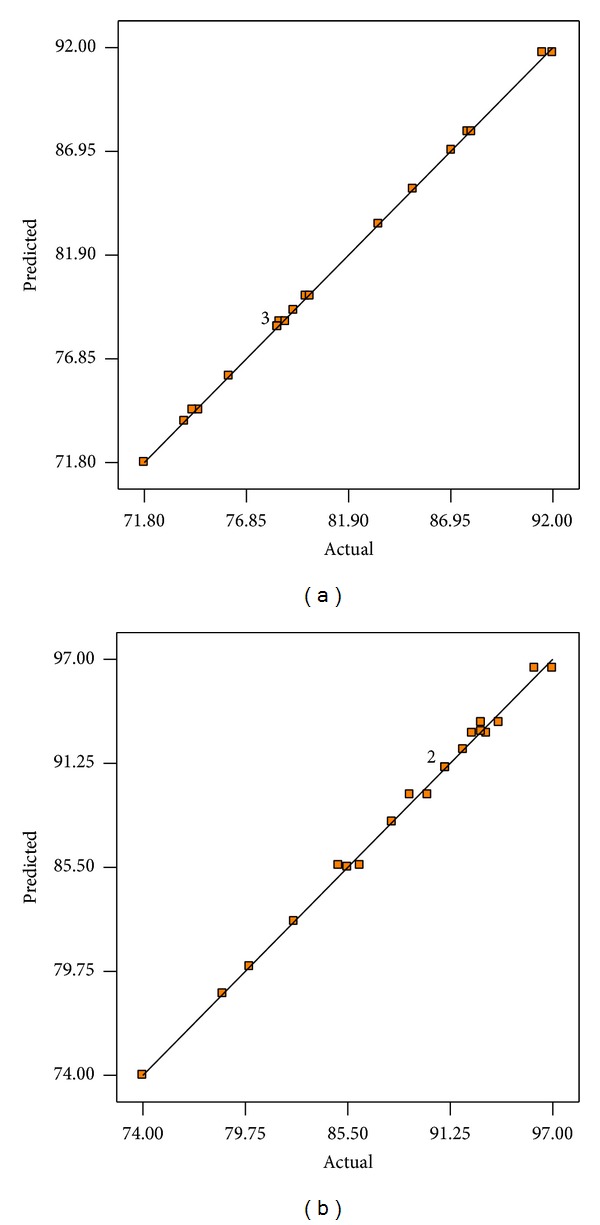
Actual versus predicted biodiesel yield using mollusks (a) and crabs (b) shells-CaO.

The relationship between predicted and experimental values of biodiesel yield for CaO prepared from MS and CS is shown in Figures 8(a) and 8(b). It can be seen that there is a high correlation (*R* ^2^ ≈ 1) between the predicted and experimental values indicating that the predicted and experimental values were in high reasonable agreement. It means that the data fit well with the model and give a convincingly good estimate of response for the system in the experimental range studied.

**Figure 9:**
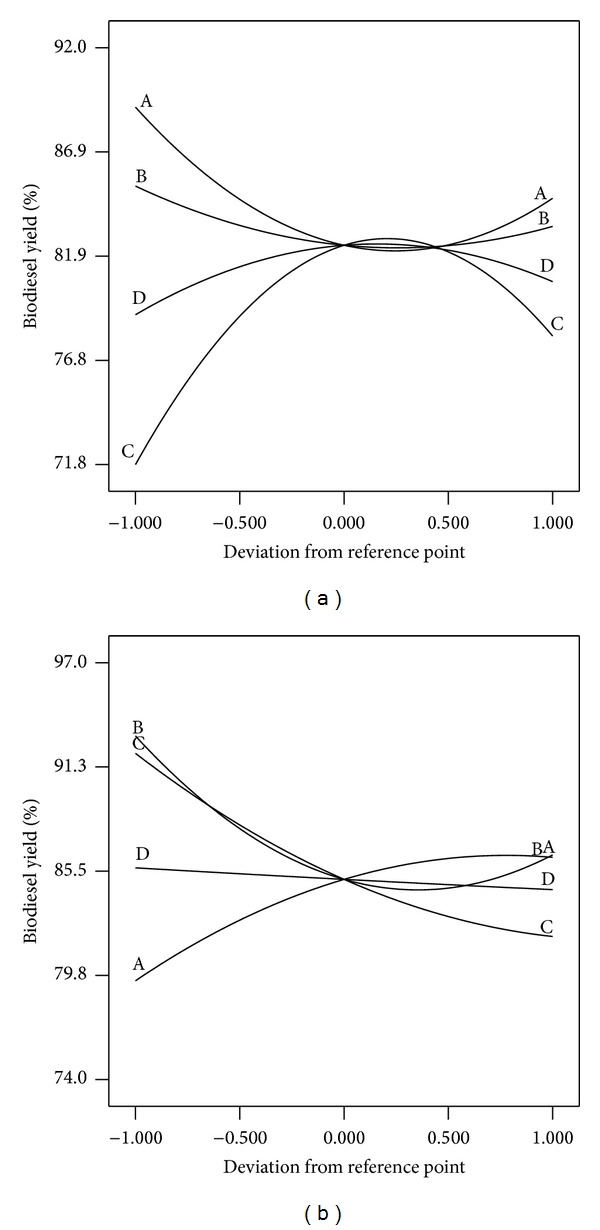
Perturbation plot for biodiesel yield in case of mollusks (a) and crabs (b) shells-CaO.

The perturbation plots in Figures 9(a) and 9(b) show the comparative effects of all independent variables on the biodiesel yield. The curvatures of the four factors from the center point confirm the statistical data obtained from analysis of variance (ANOVA, Tables 4 and 5), that is, the significance of each parameter (coefficient). In case of CaO prepared from MS (Figure 9(a)), the sharp curvature of the two factors, M : O (*A*) and reaction time (*C*), shows that the response biodiesel yield was very sensitive to these two variables. The comparatively low curvature of catalyst concentration (*B*) and mixing rate (*D*) curves shows less sensitivity of biodiesel yield towards the change in these two factors. The curvatures also confirm the data illustrated in Pareto chart (Figure 7(a)), where the increase of M : O molar ratio decreases the biodiesel yield, while the increase in reaction time increases the biodiesel yield. The sensitivity of biodiesel yield towards the four variables can be ranked in the following decreasing order reaction time > M : O > catalyst concentration > mixing rate. In case of CaO prepared from CS (Figure 9(b)), all the parameters, within the studied range, have highly significant effect on the biodiesel yield except the mixing rate (*D*) which has relatively nonsignificant effect. The curvatures also confirm the data illustrated in Pareto chart (Figure 7(b)), where the increase of M : O molar ratio increases the biodiesel yield, while the increase of reaction time, catalyst loading, and mixing rate decrease the yield, with decrease of sensitivity (reaction time > M : O ≈ catalyst concentration ≫ mixing rate). These observations are well matched to the model mathematical equations (3) and (4).

### 3.3. Response Surface Methodology

**Figure 10:**
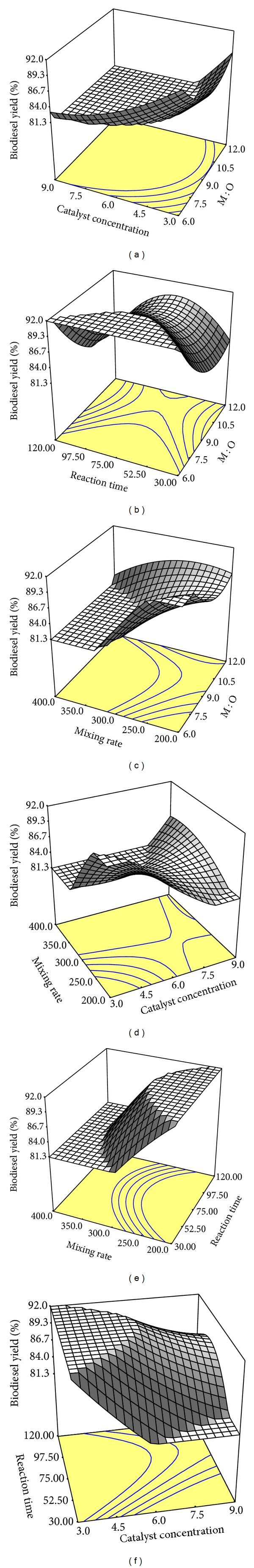
Response surface plots of biodiesel yield using CaO prepared from waste mollusks shells.

**Figure 11:**
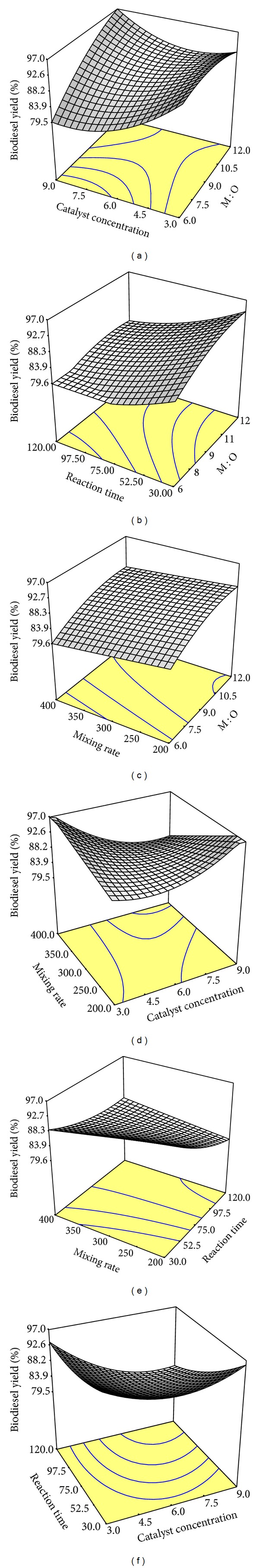
Response surface plots of biodiesel yield using CaO prepared from waste crabs shells.

Three-dimensional response surface graphical diagrams of the regression equations (3) and (4) were plotted (Figures 10 and 11) to understand the interactive relationship between the independent variables and % yield of biodiesel and determine the optimum conditions for maximum biodiesel production, using CaO prepared from MS and CS, respectively.

Figure 10(a) illustrates the effect of M : O molar ratio and the prepared MS-CaO concentration on biodiesel production at a constant reaction time of 30 min and mixing rate of 200 rpm. The increase of M : O ratio and catalyst loading decreased the biodiesel yield. The maximum yield ≈92% occurred within the range 6 : 1–6.5 : 1 M : O and 3–4.5 wt% catalyst concentration, but further increase in M : O and catalyst slightly decreased the biodiesel yield to ≈84% at 6 : 1 and 9 wt%, 89% at 12 : 1 and 6 wt%, and 81% at 12 : 1 and 9 wt%. M : O higher than the stoichiometric ratio has generally been adopted for biodiesel production. But further increase in M : O would lower the biodiesel yield as it might have dilution effect on the catalyst concentration. Glycerol would dissolve in excessive methanol and subsequently inhibit the reaction of methanol with oil and catalyst. Also the separation of glycerol would be difficult, which would consequently shift the equilibrium in the reverse direction [20].

Figure 10(b) represents the effect of M : O molar ratio and reaction time on biodiesel production at a constant catalyst concentration 3 wt% and mixing rate 200 rpm. It seems within the studied range of experiments, the increase of reaction time increased the biodiesel yield, recording ≈92% at 6 : 1–6.5 : 1 M : O and 75–93 min molar ratio. The high basicity of MS-CaO enabled transesterification in short reaction time. But the biodiesel yield slightly decreased with further increment of M : O molar ratio and time reaching ≈86% at 12 : 1 M : O and 120 min. The decrease in biodiesel production at higher reaction time might be due to the saturation of the active sites of the catalyst.

Figure 10(c) represents the effect of M : O and mixing rate on biodiesel production at a constant reaction time 30 min and MS-CaO concentration 3 wt%. Biodiesel production recorded its maximum yield ≈92% at 6 : 1–6.5 : 1 M : O and 200–250 rpm, further increment to 12 : 1 M : O and 400 rpm, decreased the yield to ≈86%.

Figure 10(d) shows the interactive effect of mixing rate and CaO concentration. It is obvious from the 3D plot the high positive interaction of these factors, at low catalyst loading and high rpm, the biodiesel yield increased recording ≈81% at 3 wt% and 400 rpm and also at high catalyst loading 9 wt% and low mixing rate 200 rpm. But at low mixing rate 200–250 rpm and catalyst loading 3 wt%, the biodiesel yield was high ≈92%.

Figure 10(e) shows the interactive effect of mixing rate and reaction time, at a constant 6 : 1 M : O and MS-CaO concentration 3 wt%. It is obvious that increasing the mixing rate and reaction time increased the yield to a certain extent recording ≈92% at 200–250 rpm and 75–93 min. Further increment of mixing rate decreased the yield recording ≈81% at 400 rpm and 30 min and 84% at 400 rpm and 120 min.

Figure 10(f) presents the interaction between reaction time and catalyst concentration. The increases of both factors decrease the biodiesel yield, recording maximum yield ≈92% at 3–4.5 wt% and 75–93 min.

From the RSM analysis it can be concluded that the maximum biodiesel production 89−92% using CaO prepared from mollusks shells MS-CaO can be achieved over a wide range of experimental parameters, at 6 : 1–6.5 M : O molar ratio, 3–4.5 wt% catalyst concentration, 75–93 min reaction time, and mixing rate of 200–250 rpm, at 60°C.

Figure 11(a) illustrates the effect of M : O molar ratio and CS-CaO catalyst loading on biodiesel production at a constant reaction time of 120 min and mixing rate 400 rpm. At low M : O ratio the increase of catalyst concentration decreased the biodiesel yield, recording ≈79.6% yield at 6 : 1 M : O and 9 wt% catalyst concentration. This can be explained by the effect of mass transfer limitation with the presence of excess solid catalyst. But at high M : O ratio, the biodiesel yield increased with increase of catalyst loading, recording maximum biodiesel yield of approximately 97% at ≈10.5 : 1–12 : 1 M : O and 7.5–9% catalyst loading (w:w). According to Son et al. [20], a molar ratio of methanol to oil M : O higher than the stoichiometric ratio has generally been adopted for biodiesel production, as methanol would promote the formation of methoxy anions on the catalyst surface, leading to a shift in the equilibrium in the forward direction, thus increasing the biodiesel yield.

Figure 11(b) represents the effect of M : O molar ratio and reaction time on biodiesel production at a constant catalyst concentration 3 wt% and mixing rate 400 rpm. It seems that at low and high M : O, the increase in reaction time decreases the yield, recording ≈80% at 6 : 1 M : O and 120 min and ≈93% at 12 : 1 M : O and 120 min but ≈97% at 12 : 1 M : O and 30 min.

Figure 11(c) represents the effect of M : O and mixing rate on biodiesel production at a constant reaction time 120 min and catalyst loading 3%. The 3D plot shows that, at low and high M : O, the increase in mixing rate decreases the yield, recording ≈97% at 12 : 1 M : O and 200 rpm but ≈93% and 80% at 400 rpm using 12 : 1 and 6 : 1 M : O, respectively.

Figure 11(d) shows that there is a negative interactive effect between these two factors, where at low mixing rate, increasing the catalyst concentration increased the biodiesel yield, recording 80 and 97% at 200 rpm using 3 and 9 wt% catalyst, respectively. But, at high mixing rate 400 rpm, the opposite occurred; increasing the catalyst loading decreased the yield, recording ≈93 and 97% at 9 and 3 wt% catalyst loading, respectively. The separation phase between hydrophilic methanol and hydrophobic oil and between the two liquid reactants and solid catalyst is generally known to be major problem, lowering the biodiesel yield in heterogeneous process. Thus, increasing the stirring rate would increase the mixing of the reactants, which would consequently promote the transesterification process, as the mass transfer of the reactants to the catalyst surface can be enhanced. Further increase in the stirring velocity would lead to increase in turbulence in the reaction mixture, which would lower the biodiesel yield. Also the increase of catalyst concentration would increase the active sites of the solid catalyst available for the transesterification process.

Figure 11(e) shows the interactive effect of mixing rate and reaction time. It is obvious that increasing the mixing rate to a certain limit 200–300 rpm, with increase of reaction time 30–50 min, yields larger amount of biodiesel, recording its maximum ≈97%. Further increase of mixing rate slightly decreased the biodiesel yield, recording ≈93% at 400 rpm and 120 min and ≈89% at 400 rpm and 30 min.

Figure 11(f) presents the negative interaction between reaction time and catalyst loading. The increase of catalyst loading and reaction time decreased the biodiesel yield to reach ≈80% at 9 wt% catalyst concentration and 120 min. The maximum biodiesel yield ≈97% occurred at 9% catalyst concentrations and 30 min reaction time and ≈93% at 3% catalyst concentrations and 120 min reaction time. According to Zhang et al. [21], the reason for the reduction in biodiesel yield with excess catalyst may be attributed to increase in viscosity of reaction mixture which might result in mass transfer limitation.

From the RSM analysis it can be concluded that the maximum biodiesel production using CaO prepared from crabs shells CS-CaO can be achieved at 10.5 : 1–12 : 1 M : O, 7.5–9% catalyst concentration, 30–50 min reaction time, and mixing rate of 200–300 rpm, at 60°C.

### 3.4. Optimization of the Transesterification Process

The optimization process was carried out to determine the optimum value of biodiesel production efficiency, using the Design Expert 6.0.7 software. According to the software optimization step, the desired goal for each operational condition (*A* M : O, *B* catalyst concentration, *C* reaction time, and *D* mixing rate) was chosen “within” the studied range. The responses (% biodiesel yield) were defined as maximum to achieve the highest performance. The program combines the individual desirability into a single number and then searches to optimize this function based on the response goal. Accordingly, the optimum working conditions and respective biodiesel production were established. The maximum predicted biodiesel yields 94 and 100% were found to be achieved at 6 : 1 and 12 : 1 M : O, 4.5 and 7.3 catalyst wt%, 82 and 30 min reaction time, and 220 and 214 rpm mixing rate, in case of using biocatalyst prepared from mollusks and crabs shells, respectively. The desirability function value was found to be 1.000 for these optimum conditions. An additional experiment was then performed to confirm the optimum results. The laboratory experiment agrees well with the predicted response values ≈96% and 98%, respectively. That indicates the process optimization based on D-optimal design of experiments was capable and reliable to optimize biodiesel production from waste cooking oil using biocatalyst prepared from MS and CS. The lower required amounts of methanol and catalyst concentrations in case of transesterification using MS-CaO relative to those using CS-CaO might be attributed to the recorded higher basicity of MS-CaO relative to that of CS-CaO.

Boey et al. [2] reported the optimum conditions for production of biodiesel (≈98%) from palm olein oil using CaO prepared from waste mud crab shells by calcination above 700°C for 2 h to be 0.5 : 1 M : O g : g, 5 wt% catalyst concentration, 2.5 h, 500 rpm, and 65°C.

Correia et al. [22] reported the production of 83.1% biodiesel yield from sunflower oil using CaO prepared from waste crabs shells calcinated at 900°C for 2 h, using the following conditions: 6 : 1 M : O molar ratio, 3 wt% catalyst concentration, 4 h, 1000 rpm, and 60°C.

Viriya-empikul et al. [1] reported 92% biodiesel yield from palm olein oil using CaO prepared from waste mollusks shells by calcination at 800°C for 2 h to be 18 : 1 M : O molar ratio, 10 wt% catalyst concentration, 2 h, and 60°C.

### 3.5. Comparing the Efficiency of the Prepared Catalyst with Chemical CaO and Novozym 435

The activity of the prepared MS-CaO and CS-CaO, using the obtained optimum conditions for production of biodiesel, was compared with that of commercially available most effective heterogeneous basic catalysts CaO and Novozym 435.

At optimum conditions of MS-CaO, 6 : 1 M : O, 4.5% catalyst wt%, 82 min, 220 rpm, and 60°C, the achieved biodiesel yield was ≈95, 83, and 40% using MS-CaO, chemical CaO, and Novozym 435, respectively. While applying the optimum operating conditions for CS-CaO, 12 : 1 M : O, 7.3 catalyst wt%, 30 min, 214 rpm, and 60°C, the biodiesel yield recorded ≈97, 78, and 35% using CS-CaO, chemical CaO, and Novozym 435, respectively. That indicates the higher efficiency of the produced MS-CaO and CS-CaO for production of biodiesel in comparison with the commercial chemical CaO and the most widely used enzyme Novozym 435.

### 3.6. Physicochemical Characterization of the Produced Biodiesel Using CaO Prepared from MS and CS

The produced biodiesel using the obtained optimum transesterification conditions of each of the prepared biocatalysts was evaluated on the basis of its fuel properties compared to Egyptian petro-diesel and international biodiesel standards as shown in Table 6. All the properties of the produced biodiesel are completely acceptable and meet most of the specifications. So it can be ranked as a realistic fuel and as an alternative to petro-diesel. This recommends the applicability of waste mollusks and crabs shells for production of cheap catalyst to produce biodiesel in a cost effective process.

The iodine value of the produced biodiesel fuel BDF was in the range of 105–108 mg I_2_/100 g oil. Iodine value is a measure of unsaturation degree. The degree of unsaturation greatly influences fuel oxidation tendency. According to EN 14214, methyl esters used as diesel fuel must have an iodine value less than 120 mg I_2_/100 g sample.

The acid value measures the content of free acids in the sample, which has influence on fuel aging. The acid value of produced biodiesel was 0.6 and 0.7 mg KOH/g, with average lowering of ≈80 and 76.67% from the used WCO, indicating better transesterification efficiency, using CaO prepared from MS than that prepared from CS. The TAN of the produced biodiesel is relatively high, but within the ASTM D6751 biodiesel standards. Candeia et al. [23] reported that the biodiesel with high TAN causes operational problems, such as corrosion and pump plugging, caused by corrosion and deposit formation.

Felizardo et al. [24] reported that density at 15°C and kinematic viscosity at 40°C are important properties, mainly in airless combustion systems because they influence the efficiency of atomization of the fuel, flow, and distribution.

The density of the produced biodiesel fuel using MS-CaO and CS-CaO recorded 0.8914 and 0.9003 g/cm^3^ compared to that of petro-diesel 0.8421 g/cm^3^. Fuel with high paraffincity has high specific gravity and low API. The produced biodiesel was characterized by higher specific gravity (0.8924 and 0.9011, resp.) and lower API value (27.06 and 25.53, resp.) compared to those of the Egyptian petro-diesel sample (0.8428 and 36.39, resp.). Therefore, volumetrically, biodiesel delivers a slightly greater amount of fuel [19].

The viscosity of the produced biodiesel recorded 5.5 and 7.6 cSt with a remarkable decrease from that of WCO of ≈89 and 84.8% through transesterification using CaO prepared from MS and CS, respectively.

The better recorded TAN, density, and viscosity of the produced biodiesel using MS-CaO would indicate better transesterification efficiency, using CaO prepared from MS than that prepared from CS. This might be attributed to the recorded higher basicity of MS-CaO relative to that of CS-CaO.

The produced biodiesel has acceptable cold flow properties pour point PP −2°C and cloud point 1°C and is characterized by lower CV (38.15–35.55 MJ/kg, resp.) relevant to that of the Egyptian petro-diesel sample (45.49 MJ/kg).

The water content of the produced biodiesel is higher than that of the Egyptian petro-diesel sample recording 312–300 and 84 ppm, respectively. But it is within the recommendable biodiesel standards, <500 ppm.

The produced biodiesel has three major advantages; it is ultralow sulfur biofuel with sulfur content of 0.003–0.006%, while petro-diesel has 0.2% sulfur. So it meets the aim of petroleum industry for ultralow sulfur diesel fuel and the biodiesel combustion will not produce large amount of sulfur oxides which lead to corrosion of the engine parts and environmental pollution. The produced biodiesel has a higher FP 148–143°C, compared to 63°C for petro-diesel. So biodiesel is much less flammable fuel than petro-diesel and hence it is much safer in handling, storage, and transport. In addition, the viscosity of the produced biodiesel 5.5–7.6 cSt is competitive to regular Egyptian standards for petro-diesel 1.6–7 cSt. Hence, no hardware modifications are required for handling the produced BDF in the existing engine.

The better qualifications of the produced biodiesel, using CaO prepared from MS than those of biodiesel produced using CaO prepared from CS, might be attributed to the higher basicity of MS-CaO than that of CS-CaO which would positively impact the transesterification reaction.

## 4. Conclusion

In the viewpoint of preparation time, energy consumption, cost of catalyst, and high quality biodiesel yield, the CaO catalyst prepared from waste mollusks and crabs shells can be recommended for biodiesel production from waste cooking oil collected from seafood restaurants. This would have a triple positive impact on environment, economic, and energy sectors.

Further work is undertaken now in EPR-Biotechnology laboratory concerning the kinetics and mechanism of the transesterification process, the reusability, and stability of the prepared biocatalysts compared to those of the chemical CaO and Novozym 435, and their effects on the biodiesel yield, its purity, and quality.

## Figures and Tables

**Table 1:** Some characteristics of the prepared biocatalysts.

Parameters	Natural MS	MS at 800°C	Natural CS	CS at 700°C
Crystal size nm	42.15	38.77	32.15	32.82
Average particle size *µ*m	11.07	9.86	10.27	8.24
*S*_BET_ m^2^/g	—	12.63	—	43.73
Pore volume cm^3^/g	—	0.046	—	0.211
Pore size nm	—	0.94	—	1.84
Basicity mmol CO_2_/g	—	52.18	—	27

**Table 2:** Chemical composition of biomass derived catalysts.

Biomass	Chemical composition (wt%)
Natural mollusks shells	C (17.35%) Mg (0.01%) Ca (26.01%) O (56.62%)
Calcinated mollusks shells at 800°C	C (3.47%) Mg (0.05%) Ca (62.32%) O (34.16%)
Natural crabs shells	C (14.44%) Na (1.1%) Mg (0.92%) Ca (29.85%) O (53.69%)
Calcinated crabs shells at 700°C	C (7.92%) Na (0.03%) Mg (0.53%) Ca (49.89%) O (41.63%)

**Table 3:** Experimental design matrix with experimental and predicted values of biodiesel yield.

Run number	Factors/levels								Biodiesel yield (wt%)			
	M : O (Molar ratio) *A*		Catalyst (wt%) *B*		Time (min) *C*		Mixing rate (rpm) *D*		CaO from mollusks shells		CaO from crabs shells	
	Levels	Actual value	Levels	Actual value	Levels	Actual value	Levels	Actual value	Experimental	Predicted	Experimental	Predicted
1	3	12 : 1	3	9	3	120	2	300	79.80	79.90	85	85.60
2	1	6 : 1	3	9	3	120	1	200	85.10	85.10	74	74.00
3	2	9 : 1	3	9	2	60	3	400	87.00	87.00	82.5	82.50
4	1	6 : 1	3	9	1	30	2	300	78.40	78.40	85.5	85.50
5	1	6 : 1	2	6	3	120	3	400	79.20	79.20	78.5	78.50
6	2	9 : 1	3	9	1	30	3	400	76.00	76.00	88	88.00
7	2	9 : 1	2	6	1	30	2	300	71.80	71.80	92	92.00
8	3	12 : 1	1	3	1	30	1	200	83.40	83.40	91	90.85
9	1	6 : 1	1	3	1	30	1	200	91.50	91.75	93	93.50
10	1	6 : 1	1	3	2	60	2	300	88.00	87.90	92.5	92.90
11	2	9 : 1	2	6	3	120	1	200	73.80	73.80	80	80.00
12	2	9 : 1	1	3	3	120	2	300	78.40	78.40	93	93.00
13	3	12 : 1	1	3	3	120	3	400	78.50	78.65	96	96.50
14	3	12 : 1	2	6	1	30	3	400	78.40	78.40	91	91.00
15	3	12 : 1	2	6	2	60	1	200	74.20	74.35	89	89.50
16	3	12 : 1	3	9	3	120	2	300	80.00	79.90	86.2	85.60
17	1	6 : 1	1	3	1	30	1	200	92.00	91.75	94	93.50
18	3	12 : 1	2	6	2	60	1	200	74.50	74.35	90	89.50
19	3	12 : 1	1	3	3	120	3	400	78.80	78.65	97	96.50
20	1	6 : 1	1	3	2	60	2	300	87.80	87.90	93.3	92.90

**Table 4:** Analysis of variance of the fitted quadratic regression model (3).

Source	SS∗	df∗	MS∗	*F* value	*P* value	Remarks
Model	671.25	14	47.95	940.12	<0.0001	Highly significant
*A*	46.97	1	46.97	920.97	<0.0001	Highly significant
*B*	3.06	1	3.06	59.98	0.0006	Highly significant
*C*	18.50	1	18.50	362.76	<0.0001	Highly significant
*D*	2.14	1	2.14	41.94	0.0013	Significant
*A* ^2^	16.46	1	16.46	322.79	<0.0001	Highly significant
*B* ^2^	2.63	1	2.63	51.52	0.0008	Highly significant
*C* ^2^	52.85	1	52.85	1036.26	<0.0001	Highly significant
*D* ^2^	5.07	1	5.07	99.36	0.0002	Highly significant
*AB*	6.80	1	6.80	133.27	<0.0001	Highly significant
*AC*	1.84	1	1.84	36.17	0.0018	Significant
*AD*	33.02	1	33.02	647.50	<0.0001	Highly significant
*BC*	8.84	1	8.84	173.38	<0.0001	Highly significant
*BD*	28.39	1	28.39	556.71	<0.0001	Highly significant
*CD*	1.08	1	1.08	21.19	0.0058	Significant
Residual	0.26	5	0.051			

Corrected total	671.50	19				

*SS: sum of squares; df: degree of freedom; MS: mean square.

**Table 5:** Analysis of variance of the fitted quadratic regression model (4).

Source	SS∗	df∗	MS∗	*F* value	*P* value	Remarks
Model	708.18	14	50.58	99.58	<0.0001	Highly significant
*A*	111.75	1	111.75	219.98	<0.0001	Highly significant
*B*	34.40	1	34.40	67.72	0.0004	Highly significant
*C*	48.63	1	48.63	95.73	0.0002	Highly significant
*D*	1.20	1	1.20	2.37	0.1845	Nonsignificant
*A* ^2^	3.92	1	3.92	7.73	0.0389	Possibly significant
*B* ^2^	15.77	1	15.77	31.05	0.0026	Significant
*C* ^2^	3.39	1	3.39	6.66	0.0493	Possibly significant
*D* ^2^	9.279*E* − 004	1	9.279*E* − 004	1.827*E* − 003	0.9676	Nonsignificant
*AB*	22.96	1	22.96	45.20	0.0011	Significant
*AC*	0.43	1	0.43	0.84	0.4021	Nonsignificant
*AD*	0.10	1	0.10	0.20	0.6720	Nonsignificant
*BC*	14.40	1	14.40	28.35	0.0031	Significant
*BD*	11.88	1	11.88	23.39	0.0047	Significant
*CD*	9.21	1	9.21	18.13	0.0080	Significant
Residual	2.54	5	0.51			

Corrected total	710.72	19				

*SS: sum of squares; df: degree of freedom; MS: mean square.

**Table 6:** Physicochemical properties of the produced biodiesel using CaO prepared from waste MS and CS compared to international standards of biodiesel and Egyptian petro-diesel standard specifications.

Test	Unit	Produced biodiesel		Egyptian perto-diesel standards	Biodiesel EN14214	Biodiesel D-6751
		Mollusks shells CaO	Crabs shells CaO			
Density at 15.56°C	g/cm^3^	0.8914	0.9003	0.82–0.87	0.86–0.9	—
Specific gravity		0.8924	0.9011	—	—	—
API		27.06	25.53	—	—	—
Kinematic viscosity at 40°C	cSt	5.5	7.6	1.6–7	3.5–5	1.9–6
Pour point	°C	−2	−2	4.5	—	—
Cloud point	°C	1	1	—	—	—
Total acid number	mg KOH/g	0.6	0.7	Nil	<0.5	<0.8
Total S	wt%	0.003	0.006	<1.2	<0.01	<0.05
Water content	ppm	312	300	1500	<500	<500
Flash point	°C	148	143	>55	>101	>130
Calorific value	MJ/Kg	38.15	35.55	>44.3	32.9	—
Iodine number	mg I_2_/100 g	105	108	—	<120	—
